# RRM2 Improves Cardiomyocyte Proliferation after Myocardial Ischemia Reperfusion Injury through the Hippo-YAP Pathway

**DOI:** 10.1155/2021/5089872

**Published:** 2021-11-25

**Authors:** Huamin Yu, Haiyan Tang, Chaochao Deng, Qing Lin, Peng Yu, Shaowen Chen, Jingming Ruan

**Affiliations:** ^1^Department of Cardiology, The First People's Hospital of Linping District, Hangzhou, China; ^2^Catheterization Room, The First People's Hospital of Linping District, Hangzhou, China; ^3^Department of Cardiovascular, Fujian Provincial Hospital South Branch, Fuzhou, China; ^4^Department of Cardiovascular, Longyan First Hospital, Longyan, China

## Abstract

**Objective:**

Ribonucleotide reductase M2 (RRM2) as an enzyme that catalyzes the deoxyreduction of nucleosides to deoxyribonucleoside triphosphate (dNTP) has been extensively studied, and it plays a crucial role in regulating cell proliferation. However, its role in ischemia-reperfusion injury (I/RI) is still unclear.

**Methods:**

SD rats were used as the research object to detect the expression of RRM2 in the myocardium by constructing an I/RI model. At the same time, primary SD neonatal rat cardiomyocytes were extracted, and hypoxia/reoxygenation (H/R) treatment simulated the I/RI model. Using transfection technology to overexpress RRM2 in cardiomyocytes, quantitative Real-Time Polymerase Chain Reaction (qRT-PCR) was used to detect the expression of RRM2, Cell Counting Kit-8 (CCK-8) assay was used to detect cell viability, and immunofluorescence staining was used to detect Ki67 and EdU-positive cells. Western blot (WB) technology was used to detect YAP and its phosphorylation expression.

**Results:**

qRT-PCR results indicated that the expression of RRM2 was inhibited in the model group, and cardiomyocytes overexpressing RRM2 can obviously promote the proliferation of primary cardiomyocytes and improve the damage of cardiac structure and function caused by I/R. At the same time, RRM2 can promote the increase of YAP protein expression and the increase of Cyclin D1 mRNA expression.

**Conclusion:**

RRM2 expression was downregulated in myocardial tissue with I/R. After overexpression of RRM2, cardiomyocyte proliferation was upregulated and the Hippo-YAP signaling pathway was activated.

## 1. Introduction

With the improvement of the national economy and the aging of the population, the number of patients suffering from cardiovascular diseases (CVD) in China is also increasing year by year. Acute myocardial infarction (AMI) has always been one of the diseases with high mortality and prevalence in CVD, and it has been increasing year by year [1, 2]. In recent years, with the development of interventional and thrombolytic therapy, the blood vessels of AMI have been opened in an early and timely manner, which has obviously reduced the mortality rate of AMI. However, cell metabolism dysfunction and structural damage will occur after myocardial I/R [3]. The nonrenewable nature of myocardial cells leads to the recognition, phagocytosis and elimination of necrotic cardiomyocytes in the ischemic area by the immune system, and their replacement by collagen tissue, while the myocardial structure, function, and morphology in the nonischemic area are remodeled and eventually develop into heart failure. Apoptosis of myocardial cells in nonischemic areas, hypertrophy, and fibrosis of myocardial tissues leads to pathological remodeling of ischemia-related tissues and myocardial dysfunction [4, 5]. Therefore, how to reduce myocardial reperfusion injury and promote myocardial cell proliferation has always been the focus of cardiovascular clinicians.

Ribonucleotide reductase (RR) was first discovered in tumors. It is a specialized enzyme that catalyzes the deoxygenation of nucleosides to dNTPs, and dNTPs are important raw materials for DNA damage repair and replication. More and more studies have suggested that the large and small subunits of RR are separated from each other in the resting state of cells, and only their specific combination with each other can perform the RR function [6]. At present, RR has been proven to regulate the proliferation and migration of tumor cells in many studies [7, 8], but there are not many studies on it in the heart. Regnier M's study found that the heart-specific overexpression of RRM2 can enhance myocardial contractility and flaccidity [9, 10]. So whether RRM2 has a similar effect in I/R, it is worth exploring.

Previous studies have proved that the Hippo-YAP signaling pathway is a crucial pathway for myocardial regeneration. Hippo cascade kinase inhibits cell proliferation and organ growth by inhibiting YAP through phosphorylation. As a transcriptional coactivator, YAP's proliferation and oncogenic activity are driven by the association of its transcription factor TEAD family, which upregulates genes that promote cell growth and inhibit cell apoptosis. When the Hippo-YAP pathway is activated, YAP is phosphorylated by serine residues and sequestered in the cytoplasm. When the Hippo pathway is not activated, YAP enters the nucleus and regulates gene expression [11, 12]. Therefore, we speculated that RRM2 can participate in the regulation of cardiomyocyte proliferation through the Hippo-YAP signaling pathway.

## 2. Methods

### 2.1. Experimental Animal

Specific-pathogen-free (SPF) grade healthy Sprague Dawley (SD) rats, clean grade healthy SD newborn rats, were provided by the animal center of Fujian Provincial Hospital. The experimental design and surgical operation engineering strictly abide by the relevant regulations of the Chinese Laboratory Animal Management Regulations. The breeding room was kept at a constant temperature of 22 ± 2°C, with alternating light and dark cycles for 12 hours. This study was approved by the Animal Ethics Committee of Fujian Provincial Hospital.

### 2.2. Construction of Rat I/RI Model

According to reports in the literature, a rat I/RI model was constructed using reversible left anterior descending coronary artery ligation. Before the operation, the rats were fasted and water for 12 hours. The next day, the rats were anesthetized by intraperitoneal injection of 2% sodium pentobarbital (2 mL/100 g). After anesthesia, they were fixed on the dissection table, and the trachea was intubated. Then, connect the small animal ventilator and connect the ECG record to observe the ST segment changes. Later, a thoracotomy was performed. After iodophor disinfection, an incision was made along the 3-4th rib of the left chest wall to expose the heart, and a threading ligation was performed at the left atrial appendage 2-3 mm (the sham operation group only performed threading but not ligation). The sign of successful I/RI construction was obvious cyanosis of the myocardial wall and ST-segment elevation in more than two leads of the ECG. After 30 minutes of ischemia, the ligature was cut and the model was successfully constructed after 2 hours of reperfusion.

### 2.3. RRM2-RNA Adenovirus Treatment

The adenovirus (Ad) RRM2 vector and the empty vector were synthesized by Shanghai Jikai Gene Chemical Company (Shanghai, China). 1 mL of the packaged adenovirus was added to normal saline to dissolve and mix, and an Ad mixture with a final volume of 200 *μ*L/mL was prepared. During the construction of the rat I/RI model, the rats in the Ad-RRM2 group were injected with 0.5 mL of the mixture, and the rats in the empty vector group were injected with an equal volume of the Ad empty vector mixture.

### 2.4. Cardiac Function Detection

The MAC 1200ST ECG analyzer and GE Logic E9 echocardiography instrument were used to detect cardiac function in rats. The rats were anesthetized by intra-abdominal injection of 2% sodium pentobarbital, then the rats were fixed on the table supine for chest depilation, and then echocardiography was performed to obtain indexes such as left ventricular end diastolic diameter (LVEDD), left ventricular end systolic diameter (LVESD), left ventricular ejection fraction (LVEF%), and left ventricular fractional shortening (LVFS%).

### 2.5. Tissue Specimen Collection

The blood and heart of each rat were collected immediately after the cardiac function test was completed. For blood collection, blood was drawn from the abdominal aorta with a 10 mL syringe and then divided into 1.5 mL centrifuge tubes after collection. After 4000 r/min centrifugation, the serum was collected and placed in a low-temperature refrigerator for storage. Then, the heart tissue was taken out, half of the heart was transected and placed in the 4% paraformaldehyde, and the rest of the heart tissue was placed in a low temperature refrigerator for later use.

### 2.6. Detection of Serum Lactate Dehydrogenase (LDH) and Creatine Kinase MB (CK-MB)

The determination of serum myocardial enzyme indexes CK-MB and LDH was carried out strictly in accordance with the instructions provided by the kits (Rongjin, Shenzhen, China), and the content of CK-MB and LDH was measured by a spectrophotometer (Life Technology, Wuhan, China).

### 2.7. H&E Staining

After the myocardial tissue was fixed with tissue fixative for 24-72 hours, it was removed from the fixative and washed with running water for 1 hour. According to the method reported in the literature, the heart tissue was sliced, and the H&E kit (Jiancheng, Nanjing, China) was used for staining. After mounting with neutral resin, the image was observed under a microscope (Thermo Fisher Scientific, Waltham, MA, USA).

### 2.8. Primary Cardiomyocyte Extraction

30 newborn SD rats were taken and soaked them in 75% ethanol for about 1 minute. After removal, the heart was quickly removed from the ultraclean workbench and placed in a petri dish filled with D-Hank solution (Thermo Fisher Scientific, Waltham, MA, USA). The heart was washed in D-Hank solution for 2-3 times and cut off the excess tissue, then transferred to a penicillin bottle and cut it into pieces, about 1 mm^3^ in size. An appropriate amount of D-Hank solution was used to wash away the remaining blood, and the supernatant was discarded after standing. Then, the tissue block was transferred to a 15 mL centrifuge tube, an appropriate amount of mixed enzymes was added into the tube at the same time, and the tube was placed in a 37°C water bath for 15 minutes. After the digestion was over, the centrifuge tube was taken out, the supernatant was discarded, an appropriate amount of complete medium was added and pipetted to mix, and then, the supernatant was transferred to a clean centrifuge tube. D-Hank solution was used to pipette the cells, and the supernatant was discarded after standing. The supernatant obtained after 3 digestions was filtered with a 200-mesh cell sieve, and the filtered supernatant was placed in a petri dish in a 5% CO_2_ 37°C incubator for differential adherence culture for 1.5 hours. After incubation, the culture dish was taken out, and the supernatant was transferred to the culture flask. Brdu solution (Thermo Fisher Scientific, Waltham, MA, USA) with a final concentration of 0.1 mmol/L was added to inhibit the growth of fibroblasts and then placed in an incubator, and the first medium change was performed after 24 hours.

### 2.9. Cell Transfection and Processing

Cardiomyocytes were seeded in a 12-well plate (5 × 10^5^ cells/well) and transfected after the cells adhered to the wall. Ad-NC and Ad-RRM2 were transfected for 8 hours. The model group cells were cultured in a hypoxic box for 2 hours, 4 hours, 6 hours, and 8 hours and then reoxygenated for 3 hours. The control group cells were cultured in a normal incubator.

### 2.10. Cell Counting Kit-8 Assay

Cardiomyocytes of each group were seeded in 96-well plates at a density of 2 × 10^3^ cells per well, and the cardiomyocytes were treated with hypoxia and reoxygenation according to the above operation. The CCK-8 kit (Ye Sen, Shanghai, China) was used to evaluate cell proliferation at each time point.

### 2.11. Immunofluorescence

Cardiomyocytes were seeded on slides at 5 × 10^5^ cells/well. After treatment, cardiomyocytes in each group were fixed with 4% paraformaldehyde. Phosphate-buffered saline (PBS) was used to wash the slides, 0.2% Triton X-100 (Jian Cheng, Nanjing, China) was used to permeate the cell membrane for 10 minutes, and then, PBS was used to wash the cardiomyocytes. Next, goat serum was used for blocking for 30 minutes. Primary antibody Ki67 (Abcam, Cambridge, MA, USA, Rabbit, 1 : 1000) was added and incubated overnight at 4°C. The next day, PBS was used to wash the slides. FITC-labeled secondary antibody was incubated at 37°C in the dark for 2 hours. After washing with PBS, 4′,6-diamidino-2-phenylindole (DAPI) dye solution (Thermo Fisher Scientific, Waltham, MA, USA) was used to stain the nuclei for 10 minutes. Next, glycerin was used to mount the slide, and the slide was placed under a fluorescent microscope to observe and take pictures.

### 2.12. EdU Staining

The transfected cardiomyocytes and control cells were cultured overnight with EdU reagent (Ye Sen, Shanghai, China). The next day, the proliferating cells were labeled with EdU DNA cell proliferation reagent. Randomly count EdU positive rate from 5 areas = EdU‐positive nucleus/DAPI‐positive nucleus.

### 2.13. Western Bolt

The total protein extraction kit was used to extract the total protein of cardiomyocytes. The bicinchoninic acid (BCA) protein quantification kit (Pierce, Rockford, IL, USA) was used to quantify the protein; then 10% sodium dodecyl sulphate-polyacrylamide gel electrophoresis (SDS-PAGE) electrophoresis were carried out and transferred the protein to a polyvinylidene fluoride (PVDF) membrane (Roche, Basel, Switzerland), the membrane in 5% skim milk at room temperature for 30 minutes was blocked, and then it was incubated with primary antibody (YAP, Abcam, Cambridge, MA, USA, Rabbit, 1 : 2000; p-YAP, Abcam, Cambridge, MA, USA, Mouse, 1 : 2000; GAPDH, Abcam, Cambridge, MA, USA, Rabbit, 1 : 5000) at 4°C overnight. Then, horseradish peroxidase-labeled secondary antibody was incubated at room temperature for 2 hours at room temperature. After incubation with enhanced chemiluminescence (ECL, Elabscience, Wuhan, China) solution, it was developed on the chemiluminescence imaging system. ImageJ software was used to analyze protein bands.

### 2.14. Total RNA Extraction and Quantitative Real-Time Polymerase Chain Reaction

TRIzol reagent (Thermo Fisher Scientific, Waltham, MA, USA) was used to extract total RNA from rat myocardial tissue and primary myocardial cells. The RNA extracted by reverse transcription using PrimeScript RT reagent kit (Thermo Fisher Scientific, Waltham, MA, USA) was cDNA, and qRT-PCR was performed using TaqMan microRNA Assay Kit (Thermo Fisher Scientific, Waltham, MA, USA). The total reaction volume was 20 *μ*L. SYBR Premix Ex Taq was used to perform qRT-PCR analysis on ABI Prism fast 7500 fluorescent quantitative PCR instrument, with GAPDH as the internal reference gene. The PCR reaction conditions were as follows: predenaturation at 95°C for 3 minutes, denaturation at 95°C for 30 seconds, annealing at 60°C for 30 seconds, extension at 72°C for 30 seconds, 30 cycles, and extension at 72°C for 5 minutes. The 2^-∆∆CT^ method was used to calculate the relative expression level. The primer sequence of the target gene after reverse transcription is shown in Table 1.

### 2.15. Statistical Analysis

Statistical Product and Service Solutions (SPSS) 20.0 statistical software (IBM, Armonk, NY, USA) were used for processing, measurement data were expressed as the mean ± standard deviation (‾*X* ± SD), a variance test was used for comparison between groups, a *t*-test was used for comparison between two groups, and *P* < 0.05 means the difference was statistically significant.

## 3. Results

### 3.1. Cardiac I/R Causes Changes in Cardiac Function and Structure

**Figure 1:**
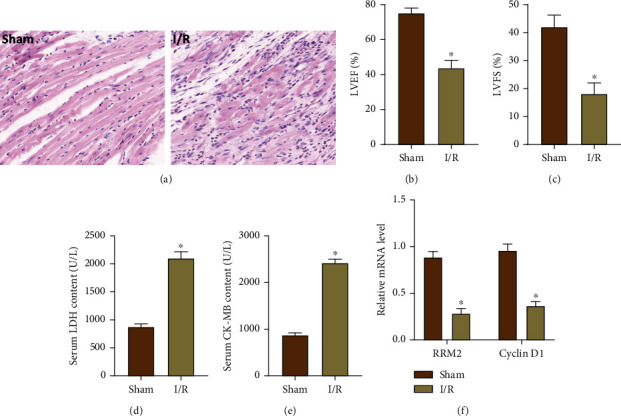
Cardiac I/R causes changes in cardiac function and structure. (a) The structural changes of heart were observed by H&E staining. (b, c) The cardiac function changes of heart were observed by echocardiography. (d, e) The serum levels of CK-MB and LDH were measured in two groups of rats. (f) The mRNA levels of RRM2 and Cyclin D1 in myocardial tissue were detected by qRT-PCR. ^∗^*P* < 0.05 vs. the Sham group.

On the first day after the establishment of the rat I/RI model, the pathological staining of cardiac tissue showed that the myocardial tissue structure of the I/R group was obviously disordered; the myocardium was broken (Figure 1(a)). At the same time, echocardiography was used to detect the cardiac function of each group of rats; the results showed that the LVEF% and LVFS% of rats in the I/R group were obviously reduced (Figures 1(b) and 1(c)). However, the heart function of rats in the sham group was normal, and the pathological staining of the heart tissue also showed a normal structure. Next, the serum LDH and CK-MB levels were detected within 24 hours after blood collection (Figures 1(d) and 1(e)). The results showed that the LDH and CK-MB levels were in the normal range in the sham group, while the I/R group LDH and CK-MB levels were obviously higher. Subsequently, we used qRT-PCR technology to detect the expression of RRM2 and Cyclin D1 from the collected heart tissue (Figure 1(f)). The results showed that the expression of RRM2 and Cyclin D1 was inhibited in the I/R group, compared with the sham group.

### 3.2. Overexpression of RRM2 in Cardiomyocytes In Vitro Can Inhibit the Reduction of Proliferation Induced by H/R

**Figure 2:**
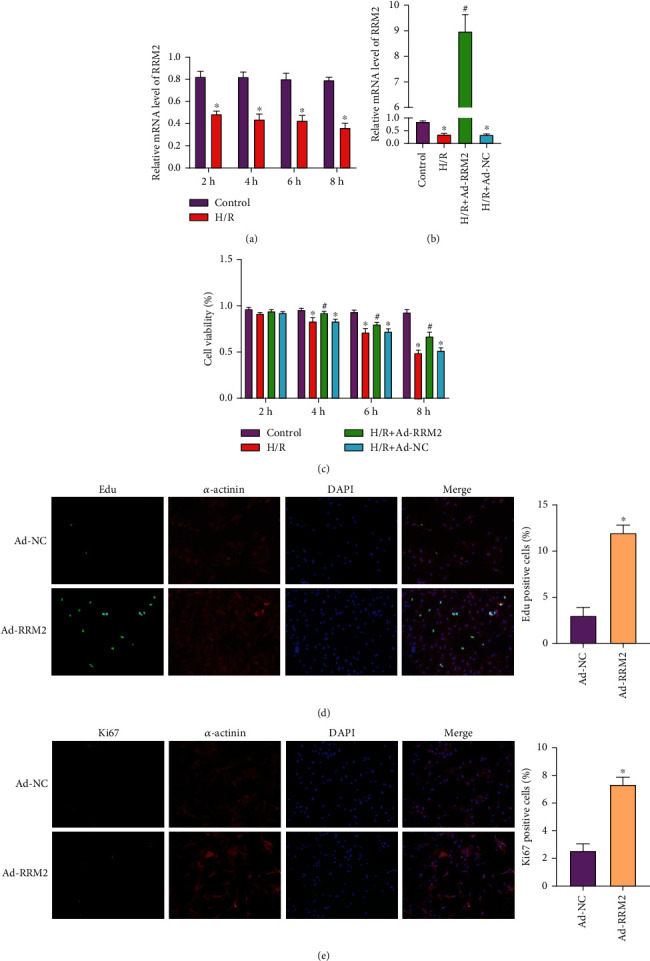
Overexpression of RRM2 in cardiomyocytes in vitro can inhibit the reduction of proliferation induced by H/R. (a) The mRNA levels of RRM2 were detected in cardiomyocytes after 2, 4, 6, and 8 h hypoxia. ^∗^*P* < 0.05 vs. the Control group. (b) qRT-PCR was used to detect transfection efficiency. (c) The cell activity of four groups was measured by CCK-8 assay. ^∗^*P* < 0.05 vs. the Control group; ^#^*P* < 0.05 vs. the H/R+Ad-NC group. (d) EdU immunofluorescence staining was used to detect cell proliferation and quantitative analysis. (e) Ki67 immunofluorescence staining was used to detect cell proliferation and quantitative analysis. ^∗^*P* < 0.05 vs. the Ad-NC group.

In order to explore the expression of RRM2 on the H/R cardiomyocyte model, the cardiomyocytes were hypoxia for 2, 4, 6, and 8 hours and then reoxygenation for 3 hours. qRT-PCR was used to evaluate the expression of RRM2 (Figure 2(a)). The results showed that the expression of RRM2 decreased obviously after H/R treatment, and the expression of RRM2 in the myocardium after H/R was time-dependent. In order to further explore the function of RRM2 in cardiomyocyte H/R, overexpression of RRM2 was used to detect its role in cell proliferation. qRT-PCR was used to evaluate the transfection efficiency (Figure 2(b)). At the same time, CCK-8 assay detected the cell viability of 4 groups, and the results showed that cardiomyocyte overexpression of RRM2 can obviously inhibit the reduction of cell viability induced by H/R (Figure 2(c)). Then, EdU and Ki67 immunofluorescence staining were used to assess the proliferation level of cardiomyocyte (Figures 2(d) and 2(e)). The staining results showed that the number of EdU- and Ki67-positive cells in Ad-RRM2 group was significantly increased compared with that in Ad-NC group.

### 3.3. Overexpression of RRM2 in Cardiomyocytes In Vivo Can Inhibit the Reduction of Proliferation Induced by I/R

**Figure 3:**
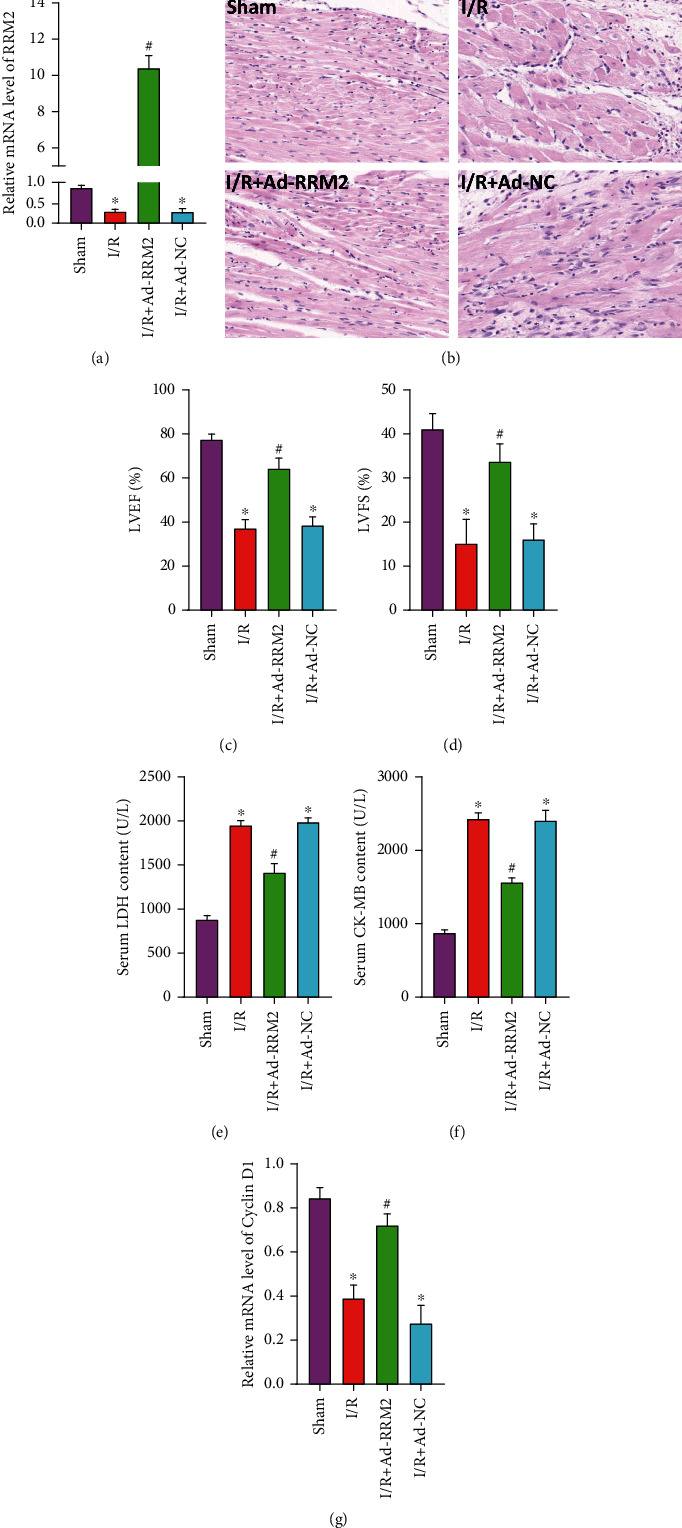
Overexpression of RRM2 in cardiomyocytes in vivo can inhibit the reduction of proliferation induced by I/R. (a) qRT-PCR was used to detect transfection efficiency. (b) The structural changes of heart were observed by H&E staining. (c, d) The cardiac function changes of heart were observed by echocardiography. (e, f) The serum levels of CK-MB and LDH were measured in four groups of rats. (g) The mRNA levels of Cyclin D1 in myocardial tissue were detected by qRT-PCR. ^∗^*P* < 0.05 vs. the Sham group; ^#^*P* < 0.05 vs. the I/R+Ad-NC group.

After the SD rat I/R model was constructed, adenovirus was injected from the pericardium. The control group was injected with Ad-NC, and the model group was injected with Ad-RRM2. One day later, we detected the expression of RRM2 in the heart of rats. qRT-PCR detection found that the expression of RRM2 in the heart of the I/R+Ad-RRM2 group was obviously increased (Figure 3(a)). At the same time, H&E staining showed that overexpression of RRM2 can alleviate myocardial tissue structural disorder, myocardial rupture, and inflammatory factor infiltration caused by I/R (Figure 3(b)). And the results of echocardiography showed that the LVEF% and LVFS% of rats in the I/R+Ad-RRM2 group were improved (Figures 3(c) and 3(d)). The levels of LDH and CK-MB in rat serum also showed a significant decrease in the I/R+Ad-RRM2 group (Figures 3(e) and 3(f)). In addition, the qRT-PCR detection of Cyclin D1 expression found that, compared with that in the I/R+Ad-NC group, the expression of Cyclin D1 in the myocardial tissue of the I/R+Ad-RRM2 group increased (Figure 3(g)).

### 3.4. RRM2 Promotes Cardiomyocyte Proliferation through the Hippo-YAP Pathway

**Figure 4:**
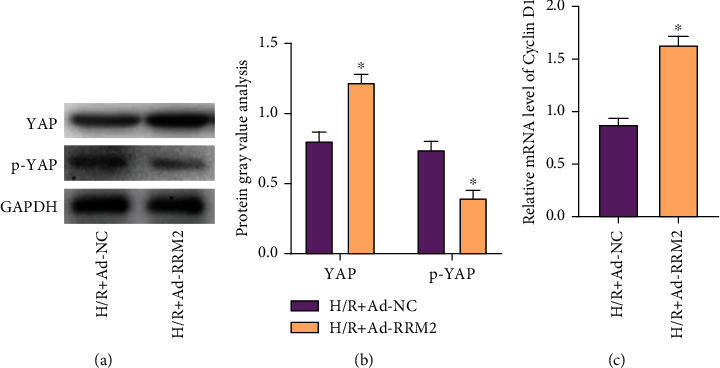
RRM2 promotes cardiomyocyte proliferation through the Hippo-YAP pathway. (a) The protein expression level of YAP and p-YAP was detected by WB. (b) Protein gray value analysis. (c) The mRNA levels of Cyclin D1 in cardiomyocyte were detected by qRT-PCR. ^∗^*P* < 0.05 vs. the H/R+Ad-NC group.

Primary cardiomyocytes were randomly divided into two groups, one of which was the H/R+Ad-NC group and the other was the H/R+Ad-RRM2 group. WB detection found that the expression of YAP in the H/R+Ad-RRM2 group was obviously increased, while the phosphorylation of YAP was reduced (Figures 4(a) and 4(b)). Subsequently, qRT-PCR detection of Cyclin D1 expression found that the Cyclin D1 expression in the H/R+Ad-RRM2 group was obviously higher than that in the H/R+Ad-NC group (Figure 4(c)).

## 4. Discussion

CVD has become the leading cause of death worldwide, and its damage to human health and economy is unmatched by other diseases [13]. Studies have shown that myocardial I/RI may lead to myocardial ischemic necrosis [14, 15]. In addition, I/RI are also common in CVD systems and patients undergoing cardiopulmonary bypass and cardiac surgery transplantation [16]. Therefore, seeking new treatment methods to improve the I/RI prognosis of patients with AMI and cardiopulmonary bypass and to avoid or reduce myocardial damage caused by organ or tissue I/RI has become a top priority for clinicians.

Studies have confirmed that the cardiomyocyte H/R model can be used as an effective method to simulate myocardial I/RI in the cardiomyocyte culture model [17]. And the duration of hypoxia is a crucial factor for the success of the H/R model. In order to determine the appropriate hypoxia time, we choose hypoxia for 2, 4, 6, and 8 hours, followed by reoxygenation for 3 hours. Cell viability decreases with the time of hypoxia. The results showed that we successfully constructed an in vitro H/R model of cardiomyocytes.

There are relatively few studies on RRM2 in the cardiovascular field. The traditional view is that RRM2 is mainly involved in the S-phase DNA synthesis process and is strictly regulated by the cell cycle [18]. Regnier M's research pointed out that RRM2 is expressed in the heart, and overexpression of RRM2 can effectively improve the cardiac function of mice after MI. Combining the two, we speculated that RRM2 also has a similar mechanism for cardiac ischemic diseases.

This study confirmed that the expression of RRM2 decreased in the myocardium of I/RI model. At the same time, LDH and CK-MB induced by I/RI are released in large quantities, inhibiting cell proliferation. Overexpression of RRM2 can obviously inhibit the levels of LDH and CK-MB in serum and promote the increase of Cyclin D1 expression. Cyclin D is a member of the cyclin family and is involved in the regulation of the cell cycle process. The synthesis of Cyclin D starts in the G1 phase and drives the G1/S transition [19]. In order to observe the effect of RRM2 on the proliferation of cardiomyocytes, we selected primary cardiomyocytes as the research object to observe their proliferation after the expression of RRM2 increased. Then, we found that the expression of RRM2 in the cardiomyocyte of H/R model also decreased. And overexpression of RRM2 can increase cell viability and promote proliferation. The results of immunofluorescence staining showed that after the expression of RRM2 increased, the proportion of Ki67 in the nucleus of cardiomyocytes increased, and the proportion of EdU also increased obviously. Ki67 is a relatively positive nuclear proliferation marker [20]. It is only expressed during the proliferation phase and is closely related to cell division. It maintains the stability of the DNA structure during cell mitosis and indicates the number of cells entering the division phase. EdU is a thymidine analogue, which can be incorporated into the replicating DNA molecule during cell proliferation. By detecting the EdU label, the DNA replication activity can be detected and accurately reflect the cell proliferation. In our study, both results indicated that overexpression of RRM2 in vitro can promote the proliferation of cardiomyocytes after H/R.

Besides, we found that RRM2 can regulate the proliferation of cardiomyocytes by influencing Hippo-YAP signal and the expression of cell cycle-related factors. The Hippo pathway and its downstream transcriptional coactivator YAP regulate organ growth and cell plasticity during animal development and regeneration. Activation of YAP in mice can promote the regeneration of adult hearts with poor regenerative capacity [21]. In mammals, the core of the Hippo pathway is cascade kinase, including LATS1 and MST1. LATS1 can bind and phosphorylate the transcription regulatory factor YAP in vitro and in vivo. At the same time, LATS1, after phosphorylation of YAP, inhibits its transcriptional regulation of cell genes by fixing YAP in the cytoplasm [22]. And we found in in vitro experiments that overexpression of RRM2 can obviously increase the expression of YAP protein, inhibit its phosphorylation expression, and promote the increase of Cyclin D1 mRNA expression. Therefore, the above results all suggested that RRM2 can be used as a potential target for the treatment of myocardial I/RI.

## 5. Conclusion

The expression of RRM2 was downregulated in the ischemia-reperfusion injury model. Overexpression of RRM2 in cardiomyocytes can promote cell proliferation, and this may be related to the activation of the Hippo-YAP pathway. This will also provide a new therapeutic target for the treatment of myocardial ischemia-reperfusion injury.

## Figures and Tables

**Table 1:** Primer sequences of quantitative reverse transcription-polymerase chain reaction.

Oligo name	Sequence (5′–3′)	
RRM2 (rat)	Forward	TGGCTGACAAGGAGAACACG
	Reverse	AGGCGCTTTACTTTCCAGCTC

Cyclin D1 (rat)	Forward	GCGTACCCTGACACCAATCTC
	Reverse	CTCCTCTTCGCACTTCTGCTC

GAPDH (rat)	Forward	CAACTCCCTCAAGATTGTCAGCAA
	Reverse	GGCATGGACTGTGGTCATGA

## Data Availability

The datasets used and analyzed during the current study are available from the corresponding author on reasonable request.
